# Sources of protein diet differentially stimulate the gut and water microbiota under freshwater crayfish, marron (*Cherax cainii*, Austin 2002) culture

**DOI:** 10.1111/1758-2229.13049

**Published:** 2022-02-07

**Authors:** Md Javed Foysal, Thi Thanh Thuy Dao, Ravi Fotedar, Sanjay Kumar Gupta, Alfred Tay, Md Reaz Chaklader

**Affiliations:** ^1^ School of Molecular and Life Sciences Curtin University Bentley WA Australia; ^2^ Department of Genetic Engineering and Biotechnology Shahjalal University of Science and Technology Sylhet Bangladesh; ^3^ ICAR‐Indian Institute of Agricultural Biotechnology Ranchi Jharkhand India; ^4^ Helicobacter Research Laboratory, Marshall Centre for Infectious Disease Research and Training, School of Biomedical Sciences University of Western Australia Perth WA Australia

## Abstract

To reduce the reliance on fishmeal (FM), other protein sources have been evaluated on cultured animals. In a 60‐days feeding trial, marrons (*Cherax cainii*) were fed a FM diet and five test diets containing 100% of plant‐based protein sources such as soybean, lupin and valorised animal‐based proteins such as poultry‐by‐product, black soldier fly and tuna hydrolysate. At the end of the trial, DNA samples from marron gut and rearing water were investigated through DNA‐based 16S rRNA gene sequencing. Plant‐based diets increased abundance for *Aeromonas*, *Flavobacterium* and *Vogesella*, whereas animal and insect proteins influenced diverse bacterial groups in the gut linked to various metabolic activities. Insect meal in the water favoured the growth of Firmicutes and lactic acid bacteria, beneficial for the marron health. *Aeromonas* richness in the gut and reared water signified the ubiquitous nature of the genus in the environment. The higher bacterial diversity in the gut and water with PBP and BSF was further supported by qPCR quantification of the bacterial single‐copy gene, rpoB. The overall results suggested that PBP and BSF can exhibit positive and influential effects on the gut and water microbial communities, hence can be used as sustainable ingredients for the crayfish aquaculture.

## Introduction

To reduce the overexploitation of already stressed wild capture fishery associated with the ecological impact of the marine environment to source fishmeal (FM) and to cater to the burgeoning protein demand for the expanded aquaculture industry, both researchers and industries have been steadily searching and evaluating various FM alternative protein sources from plant and animal‐based feedstuffs. In this context, the transformation of food waste from the poultry industry into a poultry‐by‐product meal (PBP) and seafood industry into fish protein hydrolysate or bioconversion of fish waste into insect biomass, particularly, black soldier fly (BSF) larvae offer a novel strategy to develop renewable sustainable alternative protein sources for aqua‐feed formulations in the perspectives of a circular economy. This approach will also pave the way to develop sustainable protein sources whilst reducing the conventional waste treatment such as landfilling and incineration associated with greenhouse gas emissions. PBP usually contains a higher percentage of protein, good sources of amino acids, high total digestible dry matter and energy which are comparable to FM (Galkanda‐Arachchige *et al*., 2020). Recently, BSF larvae have appeared as one of the potential alternative protein ingredients because of good nutritional profile such as protein, lipid, different functional molecules including chitin, lauric acid, bioactive peptide and different polysaccharides including choline, silkrose and dipterose (Barragan‐Fonseca *et al*., 2017; Katya *et al*., 2017; Belghit *et al*., 2019). In addition, it can valorise organic wastes or by‐products into insect biomass with a low requirement of land and water, thus representing them as a suitable candidate for promoting a circular economy in aquaculture (Chaklader *et al*., 2021a; Chaklader *et al*., 2021b).

On the other hand, though plant proteins are commonly used alternatives to FM in commercial aquafeed formulation, lack of essential amino acids and presence of anti‐nutritional factors (ANFs) discourage them from being added exclusively in the diet as these often results in inferior digestion and compromised growth of aquatic animals (Samtiya *et al*., 2020). Several studies have been conducted to minimize these shortcomings via the application of fermentation or supplementing limiting minerals (Saputra *et al*., 2019; Fan *et al*., 2021; Qian *et al*., 2021); however, their mechanism for complete substitution of FM with plant proteins on the gut health of marron (*Cherax cainii*) and other commercially cultured decapod crustaceans is yet to be explored.

To investigate the effects of protein diets on aquatic animals, researchers are now employing various molecular approaches to monitor the changes in health and immune performance. Considering the vital role of gut microbiota in nutrition, the study of gut microbial communities is immensely important to understand the impacts of various protein diets on intestinal health status, nutrients digestibility and functionality (Wang *et al*., 2018; Butt and Volkoff, 2019). Among the protein sources, soybean meal (SOY) (Catalán *et al*., 2018; Miao *et al*., 2018) and lupin (LPN) (Silva *et al*., 2011) from plant origin, PBP (Rimoldi *et al*., 2018) and tuna hydrolysate (THS) (Siddik *et al*., 2018) from animal origin, and BSF (Huyben *et al*., 2019) from insect sources have been tested vividly in various studies as alternative protein sources in aqua‐diets. These proteins also showed modulatory effects on the gut microbiota of Atlantic salmon (*Salmo salar*) (Gajardo *et al*., 2017) and juvenile barramundi (*Lates calcarifer*) (Gupta *et al*., 2020). In white shrimp (*Litopenaeus vannamei*), SOY meal (Shao *et al*., 2019) and hydrolysate from krill (Simon *et al*., 2020) have been shown to alter the gut microbiota and immune response. Similarly, in crayfishes, the dietary effect of BSF and PBP on the gut of marron has been reported (Foysal *et al*., 2019a). However, these studies have largely overlooked the potential correlation of microbial communities between gut and culture environment. As far as we are aware, no published information is available on any microbial interaction between the gut and rearing environment with different dietary protein sources for any commercially cultured decapod crustaceans, including marron.

Environmental factors shape the gut microbiota of aquatic animals. Feeding with different diets can influence diverse microbial populations in water wherein bacteria such as *Clostridium*, *Hafnia* and *Lactobacillus* have beneficial impacts on the health and immunity of aquatic animals and restoration of core gut microbiota (Nguyen *et al*., 2021). Furthermore, rearing water quality variables including organic waste accumulation can influence the bacterial interaction between the gut and the rearing environment in commercial aquaculture practices (Giatsis *et al*., 2015; Dehler *et al*., 2017; Nguyen *et al*., 2021). For instance, static water conditions with no water exchange or recirculation can transfer more bacteria from surrounding water into the gut of fish, compared to continuous flow‐through systems (Giatsis *et al*., 2015). It is therefore critical to understand the microbial interaction between the gut and rearing water for any selected aquaculture species before selecting a dietary protein source.

Recent advancements in high‐throughput sequencing technologies and computational analysis have enabled detection of microbial communities from environmental DNA (eDNA) samples. In addition, further development of databases for eDNA and metagenome prediction tools allows in‐depth analysis of microbial composition in altered environmental conditions and feeding regimes. We employed DNA‐based 16S rRNA gene sequencing to generate information about the microbial diversity and composition in the gut and rearing water with different protein diets under marron aquaculture.

## Results

### Water microbial communities are more diverse

**Fig. 1:**
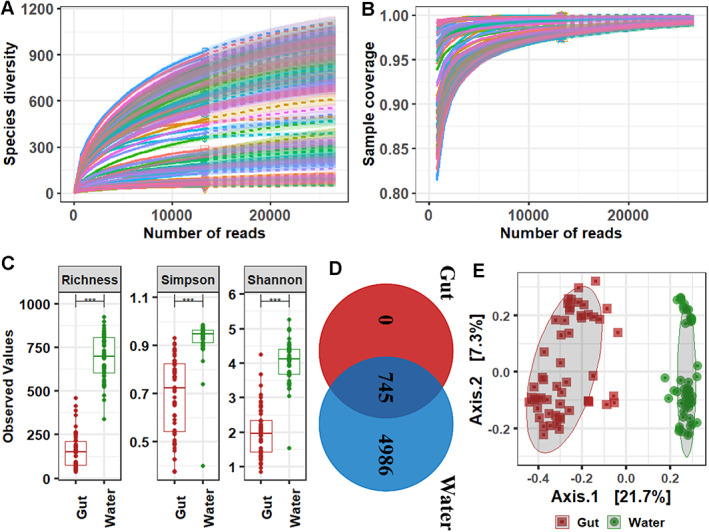
Sequence statistics and alpha‐beta diversity indices. Rarefaction curve showing the depth (A) and coverage (B) of sequences. (C) Alpha diversity, richness, Simpson and Shannon. (D) Number of shared and unique OTUs in two different environments. (E) NMDS plot showing clustering of bacterial OTUs from two different environments.

After quality filtering, a total of 4.5 M reads (41 458.4 ± 1480.6) were obtained from 108 samples. MeFiT pipeline merged the 4.3 M pair‐end reads, which made up 95.6% of the filtered sequences. The rarefaction curve revealed that each sample was sequenced at high depth, up to its saturation level to capture maximum diversity (Fig. 1A and B). Collectively, gut and water samples generated 5731 OTUs (745 shared), 26 phyla (21 shared) and 420 genera (229 shared). For the gut, 1.5 M reads (28 365.5 ± 1315.5) and 745 OTUs were obtained from 54 samples that were phylogenetically assigned into 21 phyla and 229 genera. On the other hand, water samples yielded 3.0 M reads (36 868.8 ± 1586.4) and 5731 OTUs that were classified into 26 phyla and 420 genera (Table S1). *Aeromonas* was the most abundant bacteria in the gut and water with an average rarefied read of 5070.4 ± 867.3 and 1260.8 ± 253.1 respectively; however, the gut community had significantly higher (*P* < 0.001) richness than water (Fig. S1A).

### Microbial communities are distinctly different in the gut and water

The richness, Simpson and Shannon measurements of alpha diversity were significantly higher in water, in relation to the gut (Table S1; Fig. 1C). In addition, the number of unique OTUs (4986) (Fig. 1D) and genera (191) (Fig. S1B) was found higher in water samples, compared to the gut. Beta‐ordination showed distinct clustering of bacterial OTUs wherein permutational multivariate analysis of variance (PERMANOVA) *R* ^2^ value of 0.8232 and *P*‐value of <0.0001 revealed a very different microbial diversity in the gut and water (Fig. 1E).

### Aeromonas is ubiquitous in both gut and water

**Fig. 2:**
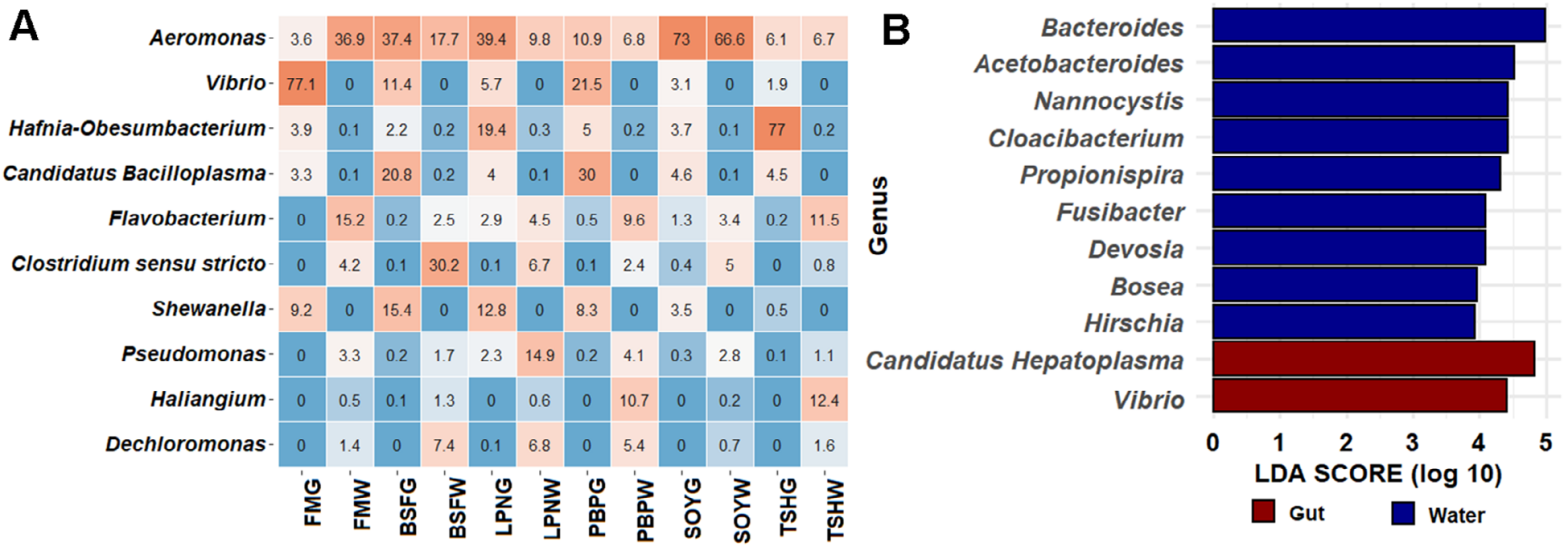
Microbial communities in the gut (G) and water (W) samples with different APS diets. A. Percentages of bacterial abundance at genus level (top 1% OTUs) in the gut and water samples. B. Differentially abundant bacteria at genus level in the gut and water with APS diets. Abbreviations: FM, fishmeal; BSF, black‐soldier‐fly meal; PBP, poultry‐by‐product meal; SOY, soybean meal; LPN, lupin meal; THS, tuna hydrolysate meal; LDA, linear discriminant analysis.

Four bacteria genera, *Acinetobacter*, *Aeromonas*, *Flavobacterium* and *Pseudomonas* had more than 1% read abundance in both gut and water samples for at least one of the treatment groups. A total of 420 genera in water including 191 unshared and 229 shared with the gut (Fig. S1B) suggesting that feeding aquatic animals with protein diets influenced a complex bacterial interaction in the water. *Aeromonas* was the most predominant bacteria in both gut and water with an average read abundance of 28.4% and 24.1% respectively; however, similarity in relative abundance between two different environments was observed only for the SOYG (73%) and SOYW (67%), and BSFG (37%) and BSFW (18%) (Fig. 2A). In addition to *Aeromonas*, higher abundance of *Vibrio* (20.1%), *Hafnia*‐Obesumbacterium (18.5%), *Candidatus* Bacilloplasma (11.2%) and *Shewanella* (8.3%) was observed in the gut microbial communities of marron (Fig. 2A). Among the genera with >1% read abundance in any of the diet group, only *Candidatus* Hepatoplasma and *Vibrio* had significantly higher abundance in the gut (Fig. 2B). Differentially abundant water bacteria were *Bacteroides*, *Acetobacteroides*, *Nannocystis*, *Cloacibacterium*, *Propionispira*, *Fusibacter*, *Devosia* and *Hirschia* (Fig. 2B).

### Protein diets modulate gut and water microbial communities

**Fig. 3:**
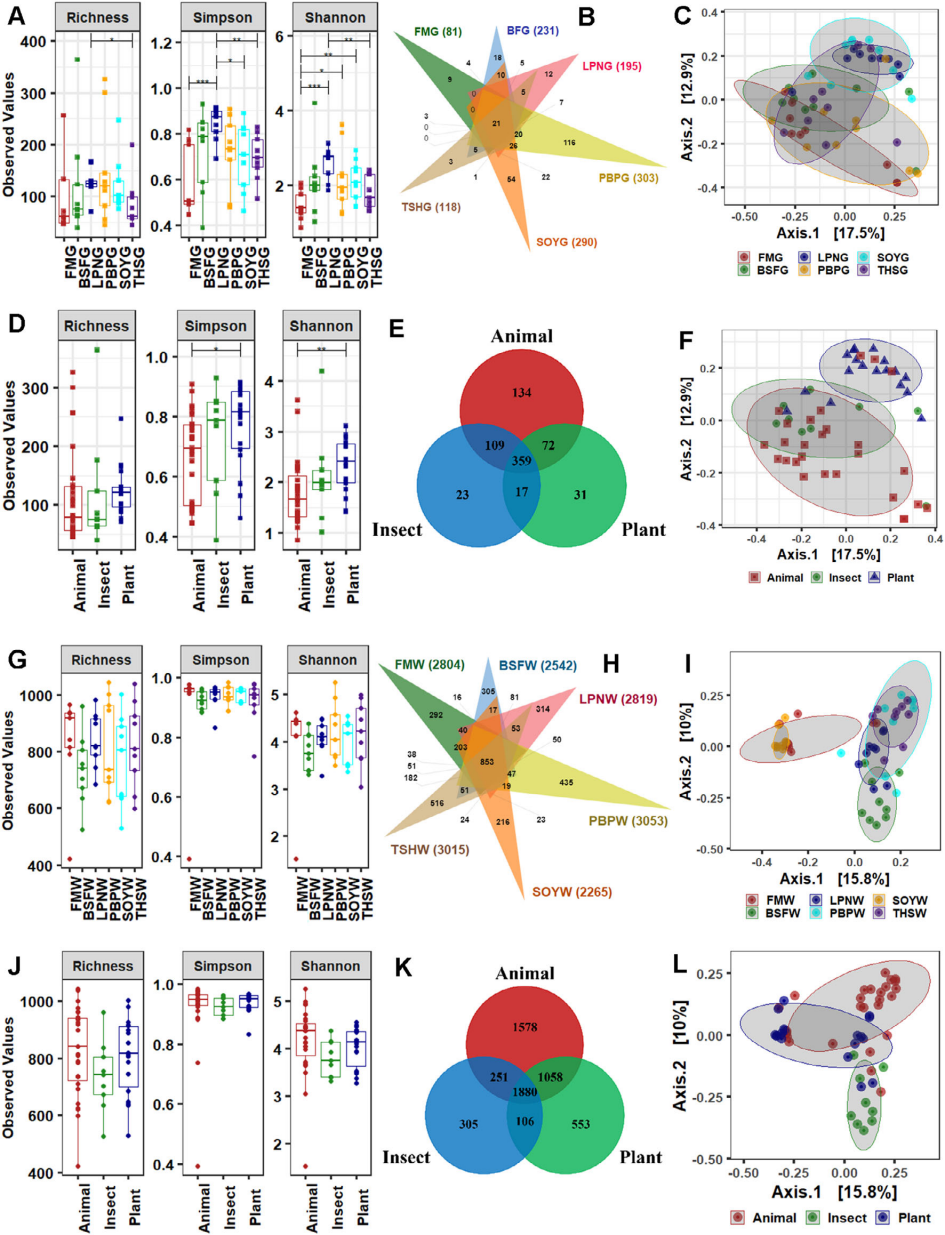
Alpha‐beta diversity indices of gut (G) and water (W) microbiota with different protein diets under marron aquaculture. Diversity in the gut, (A) Alpha diversity in terms of richness, Simpson and Shannon index; (B) Number and distributions of shared and unique OTUs; (C) NMDS plot showing clustering of bacterial OTUs. Diversity in the gut for animal, plant and insect sources, (D) Alpha diversity in terms of richness, Simpson and Shannon index; (E) Number and distributions of shared and unique OTUs; (F) NMDS plot showing clustering of bacterial OTUs. Diversity in the water, (G) Alpha diversity in terms of richness, Simpson and Shannon index; (H) Number and distributions of shared and unique OTUs; (I) NMDS plot showing clustering of bacterial OTUs. Diversity in the water for animal, plant and insect sources, (J) Alpha diversity in terms of richness, Simpson and Shannon index; (K) Number and distributions of shared and unique OTUs; (L) NMDS plot showing clustering of water bacterial OTUs. Abbreviations: FM, fishmeal; BSF, black‐soldier‐fly meal; PBP, poultry‐by‐product meal; SOY, soybean meal; LPN, lupin meal; and THS, tuna hydrolysate meal; G, gut; W, water.

The alpha diversity measurements showed higher species diversity in LPNG, than FMG. In addition, the LPNG group showed improvement in Shannon and Simpson evenness in relation to FMG and THSG (Table S2). Higher evenness for Shannon diversity was also observed for PBPG and SOYG, compared to FMG (Fig. 3A). Out of 745, only 21 OTUs (2.8%) were shared by all the dietary groups. The majority of the OTUs generated for FMG, BSFG, LPNG and THSG groups was found to be shared within and among these groups. PBPG (116) diet generated the highest unshared OTUs while only three unique OTUs were obtained from the THSG group (Fig. 3B). However, most of the unshared OTUs from the PBPG group were classified into the same taxonomic clades, mostly belonging to *Vibrio* and *C*. Bacilloplasma. The clustering of bacterial OTUs for the gut samples with different protein diets in terms of non‐metric multidimensional scaling (NMDS) is shown in Fig. 3C. The centroid analysis within the beta‐ordination demonstrated that the clustering of samples for the groups was statistically significant regarding observed dissimilarity score (*R* = 0.983) and PERMANOVA *P*‐value (<0.001). No differences in species diversity were observed while Shannon and Simpson's diversity were enriched with plant protein diets for marron, compared to animal sources (Fig. 3D). However, animal (134) diets generated the highest unshared OTUs in the gut (Fig. 3E). Alike to diet groups, the dispersion of samples for the plant, animal and insect sources was also observed to be significant (*R* = 0.722, PERMANOVA *P*‐value = 0.0223) (Fig. 3F). The relative abundance of gut bacteria showed Proteobacterial (72.8%) dominance in all diet groups (Fig. S2A). Tenericutes abundance was found higher only for the PBP and BSF feed groups, representing 42.8% and 32.4% of the read abundance (Fig. S2A). Nevertheless, Proteobacteria and Tenericutes comprised 98% of the classified reads in all groups. Several genera were limited to specific diet groups, whereas *Aeromonas*, *Candidatus* Bacilloplasma, *Hafnia* Obesumbacterium, *Shewanella* and *Vibrio* were identified from all gut samples, regardless of diets (Fig. 2B and S2B).

In the water, alpha diversity measurements found no differences (*P* < 0.05) in richness, Simpson and Shannon index among the six different groups (Fig. 3G). The number of generated OTUs (16 498) also did not differ much for six treatment groups, ranging from 984 (for the FMW) to 1190 (for the BSFW). Overall, only 5.2% (853) of the OTUs were shared by the six groups for water samples where 3.1% (516), 2.6% (435), 1.9% (314) and 1.8% (305) of the OTUs were found unique for the THSW, PBPW, LPNW and BSFW groups respectively (Fig. 3H). PERMANOVA value (*R* = 0.522, *P* = 0.032) in Bray–Curtis dissimilarity of relative abundance revealed that protein diets had a significant role in shifting microbial communities in the water used for marron culture (Fig. 3I). Though richness and diversity in the water were insignificant for the animal, insect and plant sources, the beta‐ordination found notable (*R* = 0.842, *P* = 0.001) separation of samples based on sources of protein diets (Fig. 3J–L). The ordination also showed that the OTUs for the BSFW (insect source) were distinctly different (*P* < 0.001) from other groups. Alike to gut samples, the majority of water bacteria were classified belong to Proteobacteria (59.8%). The second predominant phyla were Bacteroidetes (23.2%) and Firmicutes (16.4%) in all groups, however, the Firmicutes abundance reached 36.6% for the BSFW, and consequently, lower Proteobacterial abundance (41.6%) was observed (Fig. S3A). Similar to the gut, the dominancy of *Aeromonas* (36.6%) in water samples was identified for all groups at the genus level (Fig. S3B).

For the identification of differentially abundant bacteria, we compared the read abundance only for the lower taxonomic level. In the gut, the Kruskal–Wallis test identified eight genera with significantly different read abundance (>1%) in the six diet groups. *Aeromonas* and *Clostridium* were enriched with the SOYG diet, whereas the LPNG diet favoured the growth of *Vogesella*, *Flavobacterium* and *Pseudomonas*. In contrast, FMG, BSFG and THSG groups augmented the abundance for *Vibrio*, *Shewanella* and *Hafnia* Obesumbacterium respectively (Fig. S4). In the water, LPNW favoured the growth of *Desulfovibrio*, *Prevotella*, and *Streptococcus*, BSF augmented *Clostridium*, *Aquitalea* and *Lactobacillus*, SOYW enriched Aeromonas and Lactococcus, PBPW improved richness for *Cloacibacterium* and *Fimbriiglobus*, and THSW increased abundance for *Acidovorax* and *Stella* (Fig. S5).

### Functional features of the predicted metagenome

**Fig. 4:**
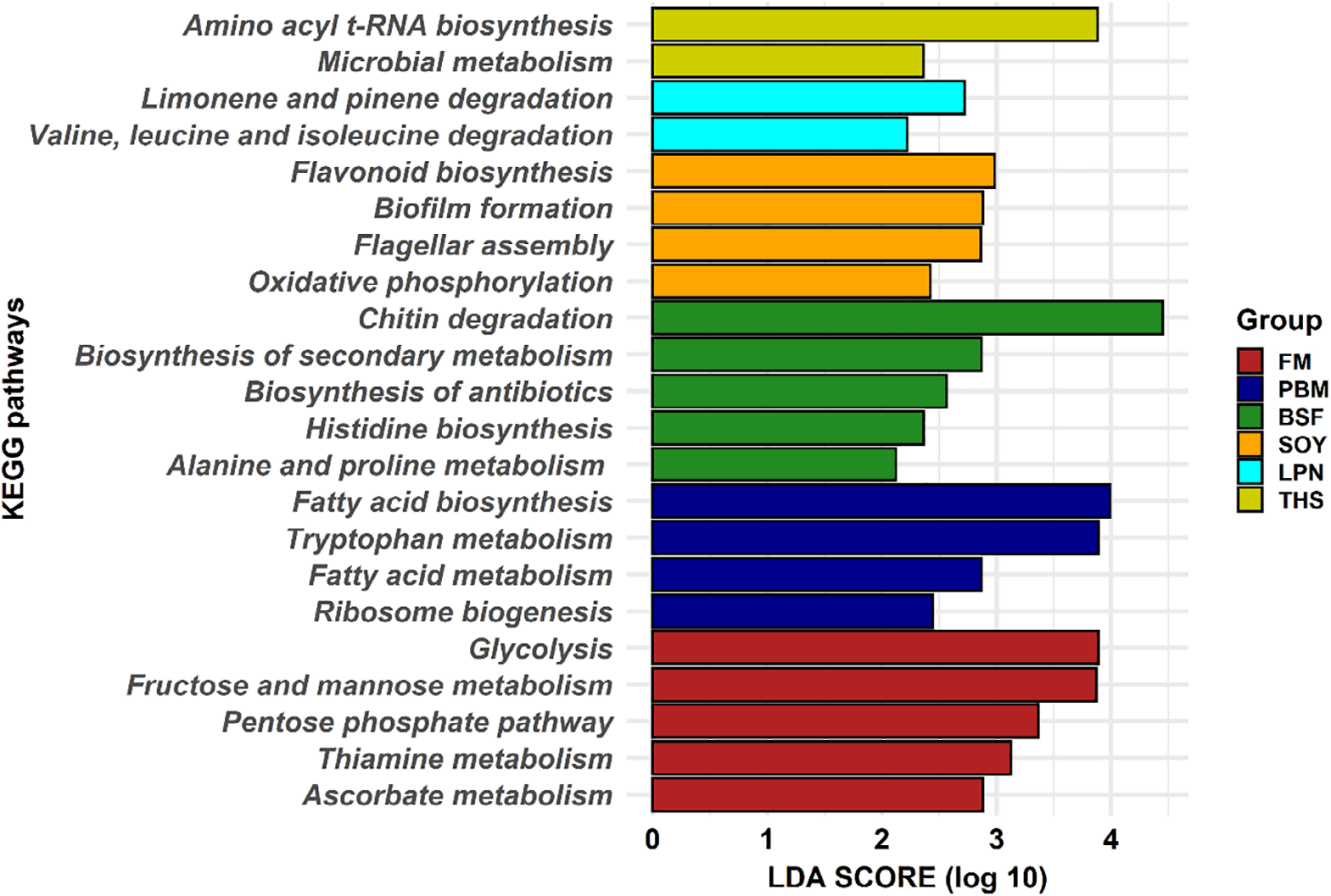
Differentially expressed pathway in the gut of marron predicted from 16S rRNA gene sequencing data using Picrust2. The statistical analysis was performed in the Galaxy server. Significantly different KEGG pathways with LDA value of ≥2.0 and *P*‐value of <0.05 are presented here. Abbreviations: FM, fishmeal; BSF, black‐soldier‐fly meal; PBP, poultry‐by‐product meal; SOY, soybean meal; LPN, lupin meal; and THS, tuna hydrolysate meal.

The Picrust2 predictions showed that animal and insect proteins FM, PBP, THS and BSF were found to associate with metabolism and biosynthesis of amino acid, fatty acid, sugar, proteins and secondary metabolites. In addition, the full‐fatted BSF larvae diet upregulated the chitin degradation pathway in the marron gut. Plant protein SOY activated flavonoid biosynthesis and some pathways linked to bacterial replication and pathogenesis while LPN was mostly involved in amino acid degradation (Fig. 4).

### Microbial quantification

Higher bacterial cell numbers were detected in marron gut fed PBP and BSF diets, significantly higher than SOY, LPN and THS while the differences were insignificant when compared with FM. Similar to 16S Illumina data, the marron fed THS diet had the lowest cell numbers. In water, the highest cell numbers were detected with the BSF diet, which was significantly higher than other experimental diets. Next to BSF, bacterial cell counts were also high in PBP and FM in relation to THS (Table 2). The *E*‐value (efficacy) of 89.6 and *R* ^2^ of 0.998 signify the reliability and reproducibility of qPCR data for all samples.

## Discussion

In recent years, microbiome analysis has become the most popular and robust tool to evaluate the impacts of dietary interventions on cultured aquatic animals. In the present study, marrons were reared under controlled environmental conditions including photoperiod, water temperature, dissolved oxygen (DO), pH and nitrogenous compounds (nitrate, nitrite and ammonia). Hence, the microbial differences in the gut and water may be primarily generated due to the differences in dietary treatments. The effects of various protein diets have been investigated on fish gut microbial communities (Gajardo *et al*., 2017; Egerton *et al*., 2020; Pérez‐Pascual *et al*., 2021; Yang *et al*., 2021); however, this is the first in‐depth study wherein impacts of different protein diets from plant sources (SOY and LNP), and processed animal protein sources (PBP, BSFL and TH) on the gut and water microbial communities, in correlations to growth performances of marron, a freshwater crayfish native to Western Australia were investigated. The microbial communities in the gut and water were distinctly different while *Aeromonas* was the only bacteria found with a read abundance of >5% in all samples. These results further reveal the ubiquitous nature of *Aeromonas* in environmental samples, as reported earlier (Janda and Abbott, 2010). To compare the results of gut microbiota with different protein diets, only two studies are currently available on crustaceans. In white shrimp (Shao *et al*., 2019), no significant difference of gut microbiota was observed with various levels of SOY meal, whereas in red swamp crayfish (Zhang *et al*., 2020), fermented SOY meal modulated gut microbiota by increasing *Bacteroides* abundance. However, no information is available so far for the effects of protein feeding on the rearing water of crustaceans. In fish, sharing of phyla and OTUs between gut and water samples were reported in Silver carp (*Hypophthalmichthys molitrix*) (Zeng *et al*., 2020) and Nile tilapia (*Oreochromis niloticus*) (Giatsis *et al*., 2015). In contrast, 2865 shared OTUs suggesting a stronger resemblance between gut and water microbial communities in marron aquaculture were obtained in this study. However, phyla and OTUs are too generalized and superficial information about the bacteria. We found that environmental bacteria *Aeromonas*, *Pseudomonas* and *Hafnia* shared most of the OTUs under marron aquaculture. This might be correlated as the stomach is very close to the mouth and oesophagus in crayfish, hence the uptake and transportation of bacteria from water into the gut can be anticipated for marron. In addition, aquaculture practices include tank culture without water exchange for marron, recirculating aquaculture system for tilapia (Giatsis *et al*., 2015), and pond culture for silver carp (Zeng *et al*., 2020) are the factors linked to bacterial differences in the water. Nevertheless, the microbial communities in the marron gut and rearing water are very different than the fishes.

In the present study, only 2% of the unshared OTUs and significant beta‐dispersion signifying the sensitivity of marron gut microbiota to protein diets were revealed. The even species distributions with LPN and highest unshared OTUs by PBP advocated gut microbiota can be selective and diversified based on sources or protein diets. Stable and consistent core microbiota with higher abundance for *Aeromonas*, *C*. *Bacilloplasma*, *Hafnia*, *Shewanella* and *Vibrio* in the gut was observed with animal and insect proteins including FM, PBP and BSF that are crucial for better gut health of marron (Saputra *et al*., 2019; Foysal *et al*., 2019a; Foysal *et al*., 2020b) and other two crayfish species, red claw (Liu *et al*., 2020) and red swamp (Shui *et al*., 2020; Zhang *et al*., 2020). Plant protein sources, LPN and SOY produced distinctly different microbiota in the gut than animal and insect sources. These variations are mainly due to the influence of plant protein on pathogenic and toxin‐producing bacteria like *Clostridium* (Cai *et al*., 2008), *Pseudomonas* (Ardura *et al*., 2013), *Flavobacterium* (Rahman *et al*., 2010), *Rheinheimera* (Chiellini *et al*., 2019) and peptidoglycan‐chitin degrading *Vogesella* (Jørgensen *et al*., 2010). Though *Clostridium butyricum* has positive impacts on crayfish gut health and immunity (Foysal *et al*., 2019b), the species identified belong to *C*. *botulinum* and *C*. *perfringens* at a 50% confidence level. We could not differentiate further due to the short read lengths (≤300 bp) and low species‐level resolution of the Illumina sequence. Previous reports on dysfunction and dysbiosis of gut microbiota due to higher inclusion level of plant protein sources and presence of ANFs (Krogdahl *et al*., 2010; Veron *et al*., 2016) might be correlated with some pathogenic bacteria in the aquatic animals. The results of dysbiosis can be further supported by the activated amino acid metabolism pathway, as gut bacteria utilize the available amino acid for their replication and assembly (Ma and Ma, 2019). Nevertheless, the overwhelming abundance of potential crayfish pathogen *Aeromonas* with SOY diet needs further investigations. In addition, further amplification of long reads is recommended for the genera having both positive and negative impacts on fish health and immunology.

In the gut, *Aeromonas*, *Candidatus* Bacilloplasma, *Hafnia* Obesumbacterium, *Shewanella* and *Vibrio* were identified as resident bacteria, which are capable of growing independently despite changes in dietary compositions. These observations are consistent with the findings of previous studies conducted on various dietary supplements (Foysal *et al*., 2019a; Siddik *et al*., 2020) and altered environmental conditions (Foysal *et al*., 2020a). Of late, studies on crayfish under different feeding regimes (Shui *et al*., 2020) and developmental stages (Zhang *et al*., 2020) have reported *C*. *Bacilloplasma* rich gut communities in the red swamp (*Procambarus clarkii*) along with *Aeromonas*, *Shewanella* and *Vibrio*, while *Hafnia* was absent in both the cases. Therefore, *Hafnia* can be used as an indicator species to differentiate between the gut microbiota of marron and red swamp crayfish. However, considering the variations in abundance of core bacteria among dietary groups, it is ambiguous that how the host plays an active role in promoting the growth of a selective core microbiota under different aqua‐diets.

Besides gut microbial alteration, feeding different protein diets also significantly altered the microbial communities in water. Higher abundance of *Lactobacillus* in qPCR and sequence data in water with BSF diet signified the influential role of this insect larva on Firmicutes and lactic acid bacteria, as reported earlier (Foysal *et al*., 2019a; Klammsteiner *et al*., 2020). With no water exchange, the chitin from an uneaten BSF‐based diet might have enhanced the colonization of *Lactobacillus* in the water and their abundance increased progressively. Similarly, BSF‐based diets improved the abundance of *Lactobacillus* in the gut and chitin was predicted as a factor that has been reported to work as a preferential substrate for lactic acid‐producing bacteria (Bruni *et al*., 2018; Terova *et al*., 2019; Chaklader *et al*., 2021a; Chaklader *et al*., 2021b). Interestingly, alike on the gut, plant diets LPN and SOY augmented *Aeromonas* (Jiravanichpaisal *et al*., 2009), *Streptococcus* (Mishra *et al*., 2018) and *Desulfovibrio* (Rath *et al*., 2018), pathogens for aquatic species. Other enriched genera including *Cloacibacterium*, *Fimbriiglobus* in PBP, *Acidovorax* and *Stella* in THS are reported ubiquitous in water; however, very little information is available about their phylogeny, nature, functions (Vasilyeva, 1985; Zhang *et al*., 2003; Kulichevskaya *et al*., 2010). This reproducible data specify a positive correlation between insect diet and beneficial bacteria for the crayfish aquaculture.

By investigating the overall gut and water microbiota, it is evident that the type and sources of dietary protein have significant impacts on gut and water microbial communities. The trial data also suggest that animal and insect sources can be used as an alternative to FM for the marron diet. The consistent results of bacterial abundance from various studies henceforth scale up the reliability, replicability and reproducibility of data. Since the impacts of feed and water have significant impacts on shaping the gut microbiota of aquatic species (Giatsis *et al*., 2015), a mixture of animal and insect diets (FM + BSF or PBP + BSF) could be a potential diet of interest for other crayfish aquaculture. However, the variation of feeding in different life cycles of crayfish (Zhang *et al*., 2020), the shift of microbiome from juvenile to adult phage (Cicala *et al*., 2020) and limitations of Illumina sequences and sequence databases to generate species‐level information (Alberdi *et al*., 2019) are some of the major concerns that uphold the importance of scaling up the sequencing‐based aquaculture nutrition studies to create a solid framework from where major ecological conclusions can be drawn. Nevertheless, based on the study design, the number of replicates used, the volume of data generated and comparative analysis performed in this study, we endorse the consistency, reliability and replicability of the eDNA results of crayfish.

## Conclusion

Dietary protein sources from PBP and BSF larvae meal improved the gut and water bacterial diversity including some beneficial bacteria, suggesting PBP and BSF larvae meal‐based protein sources could be beneficial for marron culture. However, it is recommended to conduct further research to investigate the potential components in PBP and BSF that could be responsible for influencing the microbial community both in the diet and water. Further research is recommended to separate the role between feed and faecal matter in deciding the bacterial dynamics in marron culture.

## Experimental procedures

### Experimental set‐up and animal husbandry

A total of 170 marrons (71.2 ± 0.4 g) were procured from Blue Ridge Marron Farm, Manjimup, Western Australia (34.2019 S, 116.0170 E). Marrons were transported in live conditions to Curtin Aquatic Research Laboratories (CARL), and distributed randomly into 18 tanks (nine marrons per tank, X3 replicates). Each tank of 200 L capacity was filled with 150 L underground freshwater (Fig. S4). Marrons were acclimatized for 7 days before starting feeding trial. Fixed temperature (20°C) and constant aeration were maintained. The pH and DO of water were monitored using a portable digital C/mV/pH meter (CyberScan pH 300; Eutech Instruments, Singapore) and digital DO meter (YSI55; Perth Scientific, Australia) respectively. Considering the aims of the study, no water exchange was performed during the trial. Uneaten feedstuffs and faecal wastes were removed once a week using a filter net.

### Feed formulation and feeding trial

Six isoproteic, isolipidic and isocaloric diets containing FM, BSF meal, LPN meal, PBP, SOY and THS meal were prepared (Table 1). The ingredients were supplied by Glenn Forrest, Western Australia and after feed formulation the test diets were also prepared by the same company. Proximate compositions of diets were determined as per the method of the Association of Official Analytical Chemists, AOAC (AOAC, 2005). Marrons were fed everyday afternoon at 1.5% of the total biomass in the tank.

**Table 1:** Ingredients and proximate composition of final diets (%).

Ingredients	FM	PBP	BSF	SOY	LPN	THS
Fishmeal	46	0	0	0	0	0
Poultry by product	0	42	0	0	0	0
Soybean	0	0	0	62	0	0
Black soldier fly larvae	0	0	33.6	0	0	0
Lupin	0	0	0	0	70	0
Tuna hydrolysate	0	0	0	0	0	27
wheat (10 CP)	30	34.5	33.4	12	7	35
Corn/wheat starch	11	11	11	10	11	11
Cholesterol	0.5	0.5	0.5	0.5	0.5	0.5
Canola oil	2	1.5	0	4	2	0
Cod liver oil	3	2	0	5	2.5	0
Vitamin premix	0.3	0.3	0.3	0.3	0.3	0.3
Vitamin C	0.1	0.1	0.1	0.1	0.1	0.1
Dicalcium phosphate	0.1	0.1	0.1	0.1	0.1	3
Lecithin‐Soy (70%)	3	3	3	3	3	4
Barley	4	5	5	3	3.5	19
Casein	0	0	13	0	0	0
**Total**	100	100	100	100	100	100
Proximate composition of the final diet						
Crude protein	30.5	30.5	30.0	30.4	30.2	30.7
Crude lipid	12.5	12.8	12.5	12.8	12.6	12.6

**Table 2:** Bacterial cell numbers in the gut and water after trial.

Groups (gut)	Bacteria (rpoB, cells g^−1^)	Groups (water)	Bacteria (rpoB, cells ml^−1^)
FMG^b^	1.08 ± 0.3 × 10^5^	FMW^c^	3.09 ± 0.3 × 10^4^
PBPG^a^	2.01 ± 0.4 × 10^6^	PBPW^b^	8.72 ± 0.3 × 10^4^
BSFG^a^	1.98 ± 0.2 × 10^6^	BSFW^a^	1.2 ± 0.8 × 10^5^
SOYG^b^	1.2 ± 0.5 × 10^5^	SOYW^c^	3.78 ± 0.8 × 10^4^
LPNG^bc^	0.98 ± 0.3 × 10^5^	LPNW^c^	2.98 ± 0.3 × 10^4^
THSG^c^	8.08 ± 0.3 × 10^4^	THSW^d^	9.18 ± 0.4 × 10^3^

Group with same superscript letters in the column are not significantly different.

### Sampling

Extraction of DNA from water samples was done following the method described earlier (Hinlo *et al*., 2017; Jeunen *et al*., 2019). Water samples (200 ml/tank) were collected at days 58, 59 and 60 in the sterile plastic bottle, centrifuged at 8000 rpm for 10 min, followed by membrane filtration using 0.2‐μm polycarbonate filters. The filters were then cut into small pieces (~1 mm) and transferred into 2‐ml Eppendorf tubes. Fifty microliters of DEPC‐treated water was added to each tube followed by 6‐cycles of homogenization in FastPrep‐24 5G (MP BIO, USA) for 40 s at 6.0 m s^−1^ with sterile beads. For gut microbiota analysis, 54 marrons were collected from 18 tanks (three marrons/tank) at the end of the trial. Marron gut with mucosa and pellets from water was transferred into 2‐ml Eppendorf tubes and homogenized following the methods mentioned above for water. Approximately, 200 mg of samples were used for DNA extraction.

### DNA extraction, PCR amplification and 16S rRNA gene sequencing

DNA extraction was performed using DNeasy Blood and Tissue Kit (Qiagen, Hilden, Germany) following the manufacturer's instructions. The quality of DNA was assessed in NanoDrop Spectrophotometer 2000 cc (Thermo Fisher Scientific, USA). An even concentration of 50 ng μl^−1^ was used to amplify the V3–V4 bacterial hypervariable regions according to Illumina 16S metagenomic sequencing protocol (Part # 15044223 Rev. B). PCR amplification was performed with Hot Start 2× Master Mix (New England BioLab, USA) for 50 μl final volume. Thirty‐five cycles of amplification (Bio‐Rad Laboratories, USA), beads clean‐up, amplicon meta‐barcoding, pooling and 2 × 300–base pair paired‐end MiSeq sequencing (Illumina, San Diego, CA, USA) were performed according to Illumina 16S metagenomic sequencing protocol (Part # 15044223 Rev. B).

### Sequence data processing

TrimGalore (v0.6.6) (https://www.bioinformatics.babraham.ac.uk/projects/trim_galore/), FastQC (Andrews, 2010) and NGmerge (Gaspar, 2018) were used for trimming, quality checking and merging of reads respectively. Micca (v1.7.2) was used for the filtering of merged sequences and *de novo* greedy clustering into operational taxonomic units (OTUs) at 99% similarity threshold level. Phylogenetic assignment of the representative OTUs was performed against SILVA 1.32 release (Quast *et al*., 2012). The rarefaction depth value was set to 17 796 bp and subsequent calculations of alpha‐beta diversity were performed in QIIME (v1.9.1) (Caporaso *et al*., 2010) and R packages (R Core Team, 2021).

### Downstream bioinformatics

Alpha diversity of samples was calculated in terms of richness, Simpson and Shannon index in and phyloseq (McMurdie and Holmes, 2013) R package. Beta‐ordination as NMDS was calculated based on Bray–Curtis dissimilarity of relative abundance. Relative abundance of bacterial OTUs at phyla and genus level was calculated in ampvis2 (Andersen *et al*., 2018) R package. Functional features of the metagenome were predicted using Picrust2 in support of KEGG pathway (Douglas *et al*., 2020).

### Quantification of total bacteria

The DNA extracted from 200 mg homogenized gut samples and 1 L (1000 ml) was used to quantify total number of bacteria using quantitative polymerase chain reaction (qPCR). For the quantification, bacterial single‐copy housekeeping gene rpoB (rpoB4f and rpoB2r) was used as primers for qPCR, as reported earlier (Ogier *et al*., 2019). The qPCR reactions were performed by preparing 25 μl of final master mix containing 12.5 μl QuantiFast SYBR Green PCR Kit (Qiagen), 1 μl of each primer, 1 μl of DNA and 9.5 μl of RNase‐free water. Forty cycles of qPCR reactions were performed in CFX96 Real‐Time PCR Detection System (BioRad Laboratories, USA) under the following conditions: initial denaturation at 95°C for 5 min, followed by denaturation for 10 s at 95°C, 30 s (annealing) at 60°C, 30 s (extension) at 72°C for 40 cycles. All samples were run in triplicate reactions and absolute quantification was performed following the method described by Rao *et al*. (2013).

### Statistical analysis

Statistical analysis was performed in R statistical environment (v3.6.1) (R Core Team, 2021). PERMANOVA was measured in vegan R packages (Dixon, 2003). Wilcoxon rank test (for two groups) and Kruskal–Wallis (>two groups) followed by Bonferroni correction were employed to identify the differentially abundant bacteria. Linear discriminant analysis effect size was used to identify differentially expressed pathways in different groups (Segata *et al*., 2011). At all stages, a *P*‐value of 0.05 was considered statistically significant and annotated as *P* < 0.001 (***), *P* < 0.005 (**) and *P* < 0.05 (*).

## Funding

The laboratory trial and sequencing support were obtained from the Research Training Program (RTP) Stipend Scholarship, funded by the Department of Education, Skills and Employment, Australian Government to Md Javed Foysal (No. 19059800‐Curtin).

## Data Availability

The raw sequence files are currently available in the National Centre for Biotechnology Information (NBCI) database under the BioProject accession PRJNA749331.

## Supporting information


**Fig. S1.** (A) The read abundance of *Aeromonas* in the gut and water. (B) Number of shared and unique genera in the gut and water.
**Fig. S2**. Relative abundance (in gut) of bacterial OTUs. (A) At phylum level. (B) At genus level (top 12).
**Fig. S3**. Relative abundance (in water) of bacterial OTUs. (A) At phylum level. (B) At genus level (top 12).
**Fig. S4**. Differential abundance of bacteria at genus level in the gut of marron fed different protein diets. Genera with more than 1% of read abundance in any of the group were used for statistical analysis.
**Fig. S5**. Differential abundance of bacteria at genus level in the water under marron aquaculture fed different protein diets. Genera with more than 1% of read abundance in any of the group were used for statistical analysis.
**Fig. S6**. An outline of experimental set‐up and methodologies used in present study.
**Table S1**. Major diversity index for microbial communities in the gut and water
**Table S2**. Major diversity index for microbial communities in the gut with six different diets.
