# Supplementary material for: Sources of protein diet differentially stimulate the gut and water microbiota under freshwater crayfish, marron (Cherax cainii, Austin 2002) culture
Source: Environ Microbiol Rep. 2022 Feb 7;14(2):286–98. doi: 10.1111/1758-2229.13049 (PMC9303337; doi:10.1111/1758-2229.13049)
Supplement: Supplementary file 1 — Fig. S1. (A) The read abundance of Aeromonas in the gut and water. (B) Number of shared and unique genera in the gut and water. Fig. S2. Relative abundance (in gut) of bacterial OTUs. (A) At phylum level. (B) At genus level (top 12). Fig. S3. Relative abundance (in water) of bacterial OTUs. (A) At phylum level. (B) At genus level (top 12). Fig. S4. Differential abundance of bacteria at genus level in the gut of marron fed different protein diets. Genera with more than 1% of read abundance in any of the group were used for statistical analysis. Fig. S5. Differential abundance of bacteria at genus level in the water under marron aquaculture fed different protein diets. Genera with more than 1% of read abundance in any of the group were used for statistical analysis. Fig. S6. An outline of experimental set‐up and methodologies used in present study. Table S1. Major diversity index for microbial communities in the gut and water Table S2. Major diversity index for microbial communities in the gut with six different diets. [file EMI4-14-286-s001.docx]

**Supplementary data**

**Sources of protein diet differentially stimulate the gut and water microbiota of freshwater crayfish, marron (*Cherax cainii*, Austin 2002)**

Md Javed Foysal ^1,2*^, Thi Thanh Thuy Dao ^1^, Ravi Fotedar ^1^, Sanjay Kumar Gupta ^3^, Alfred Tay ^4^, Reaz Chaklader ^1^

**Affiliations**

^1^ School of Molecular and Life Sciences, Curtin University, Bentley, WA, Australia

^2^ Department of Genetic Engineering and Biotechnology, Shahjalal University of Science and Technology, Sylhet, Bangladesh

^3^ ICAR-Indian Institute of Agricultural Biotechnology, Ranchi, Jharkhand, India

^4^ Helicobacter Research Laboratory, Marshall Centre for Infectious Disease Research and Training, School of Biomedical Sciences, University of Western Australia, Perth, WA, Australia

^*^Corresponding author:

Md Javed Foysal

School of Molecular and Life Science, Curtin University, Bentley, WA, Australia

Email address: [mjfoysal-geb@sust.edu](mailto:mjfoysal-geb@sust.edu) or [mdjaved.foysal@postgrad.curtin.edu.au](mailto:mdjaved.foysal@postgrad.curtin.edu.au)

Telephone: +61 8 9266 4508: mobile +61451404337

ORCID: <https://orcid.org/0000-0002-2064-8897>


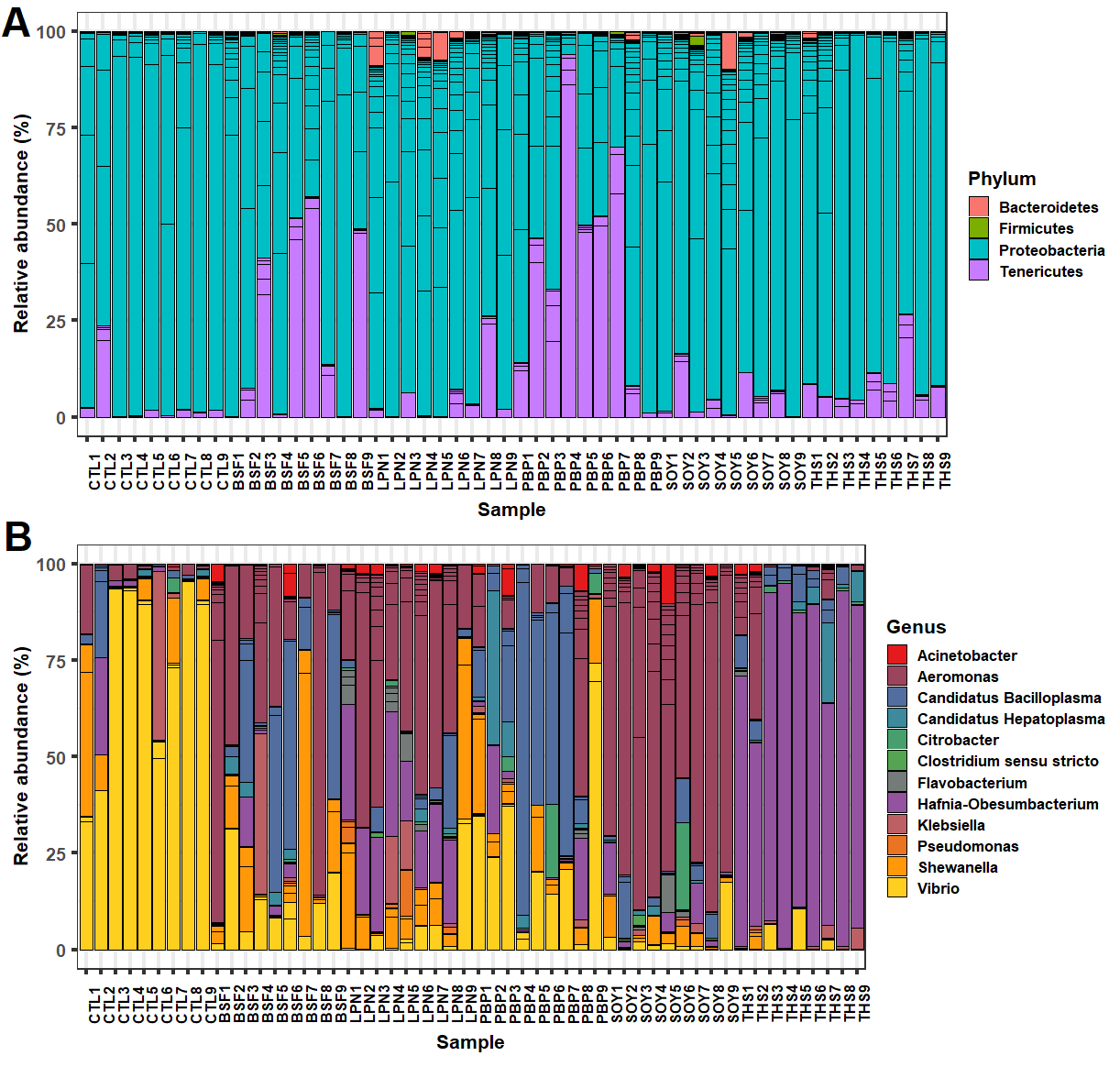


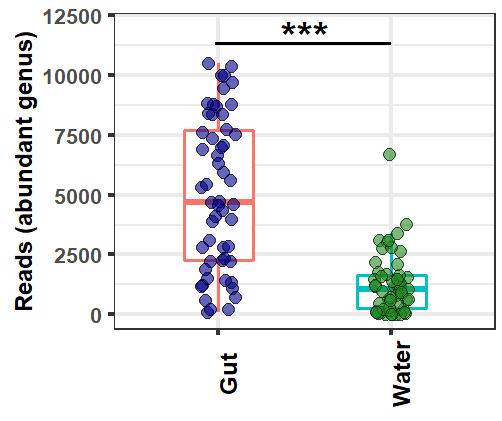


**A**


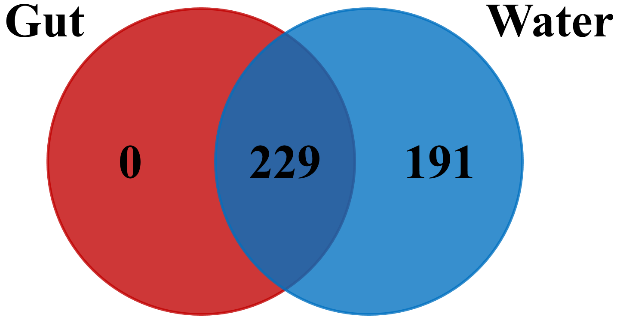


**B**

**Fig. S1**. (A) The read abundance of *Aeromonas* in the gut and water. (B) Number of shared and unique genera in the gut and water.

**Fig. S2**. Relative abundance (in gut) of bacterial OTUs. (A) At phylum level. (B) At genus level (top 12).


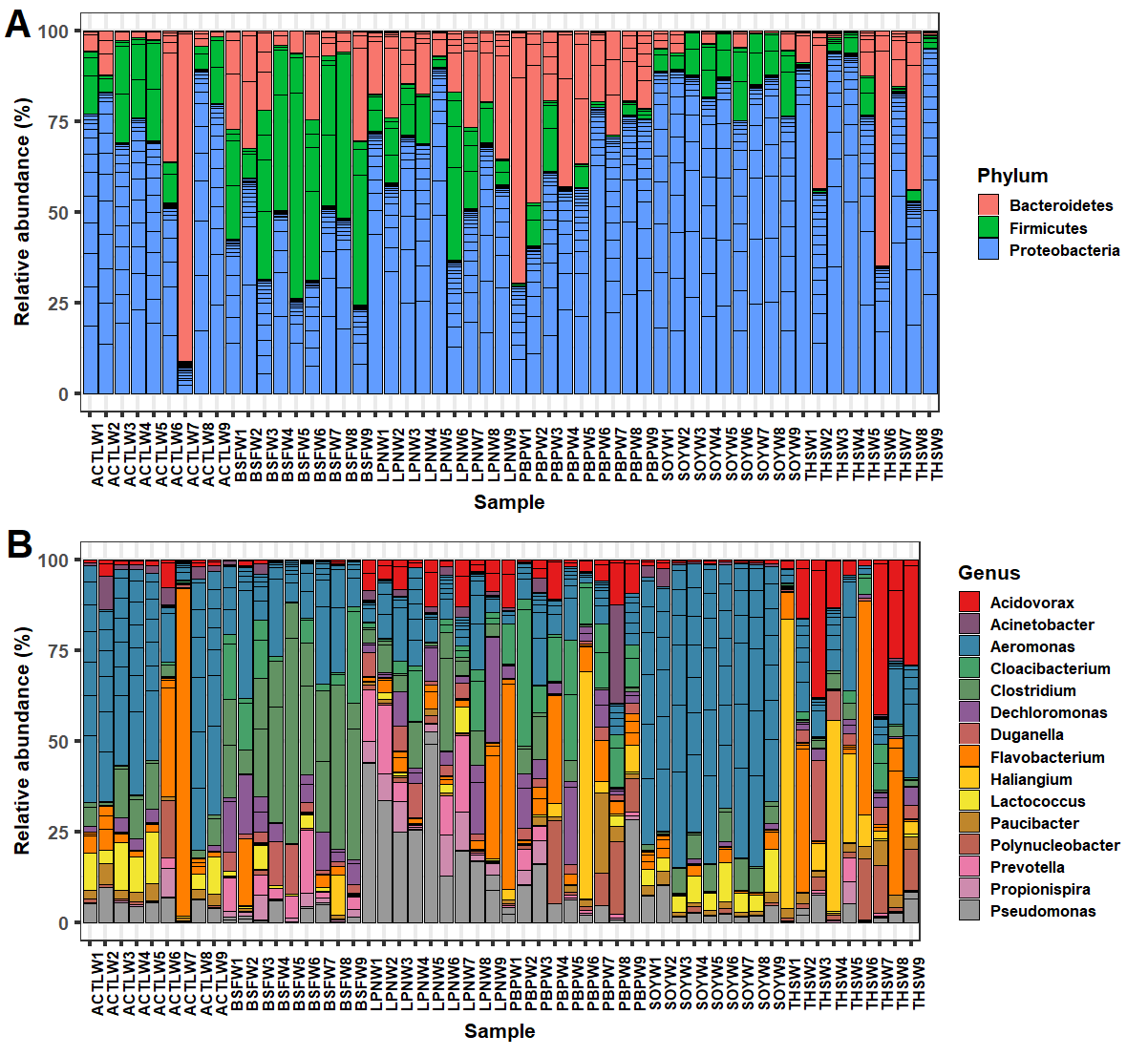
**Fig. S3**. Relative abundance (in water) of bacterial OTUs. (A) At phylum level. (B) At genus level (top 12).


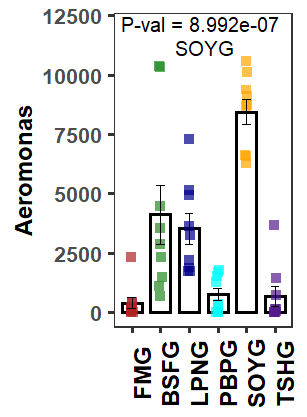

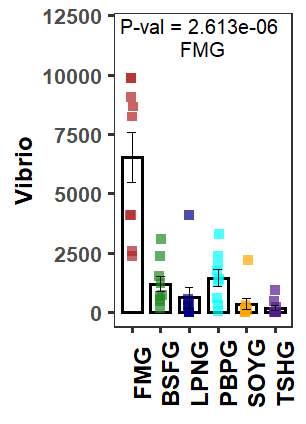

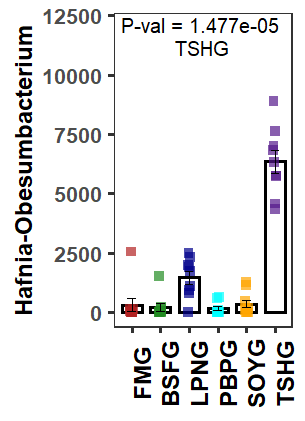

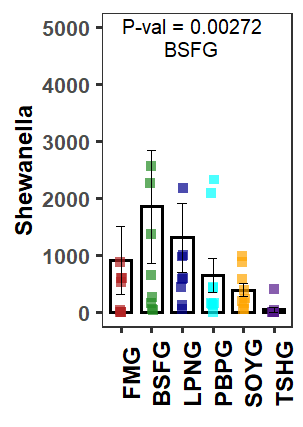

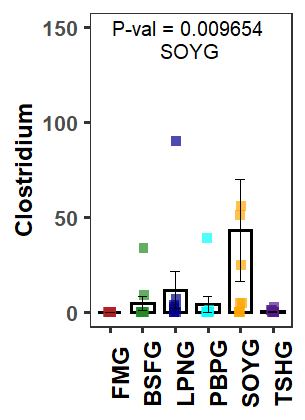

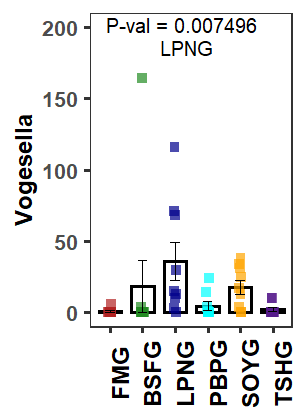

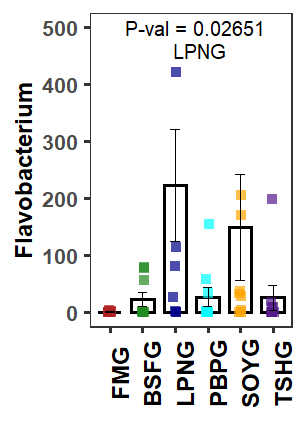

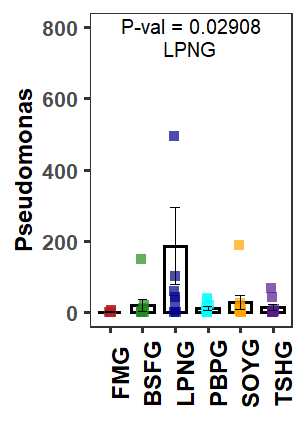


**Fig. S4**. Differential abundance of bacteria at genus level in the gut of marron fed different protein diets. Genera with more than 1% of read abundance in any of the group were used for statistical analysis.


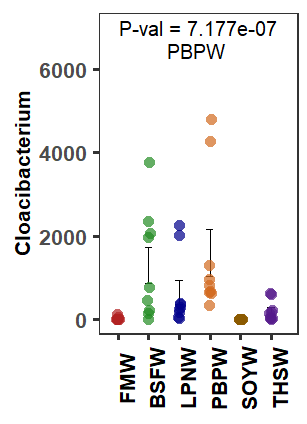

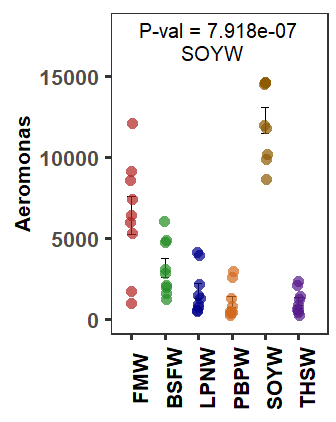

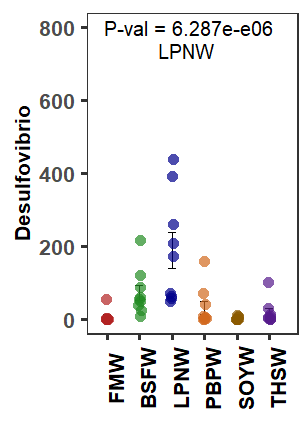

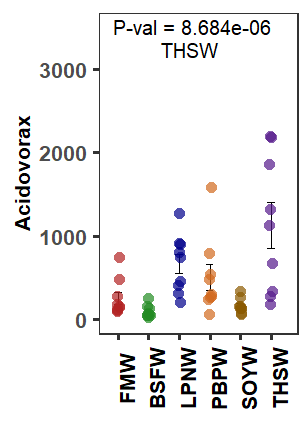

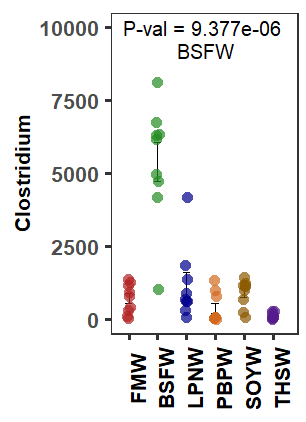

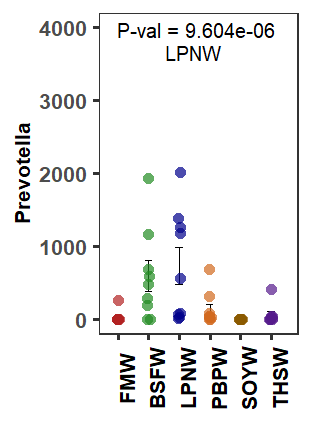

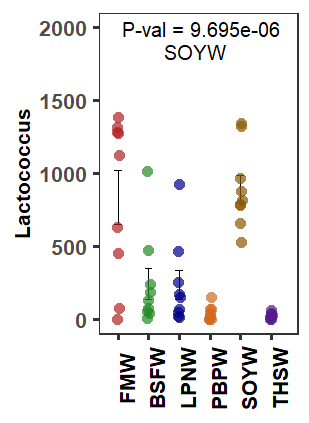

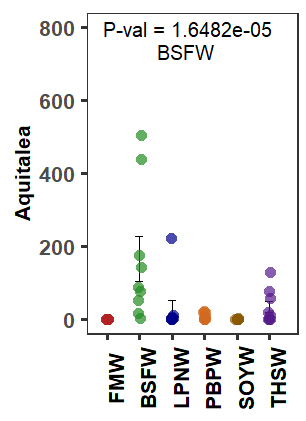

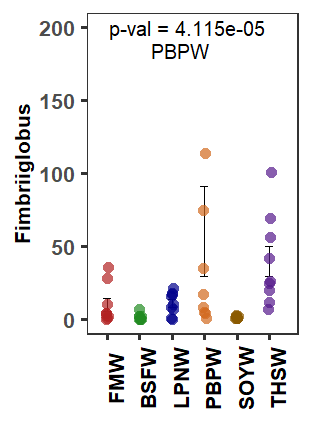

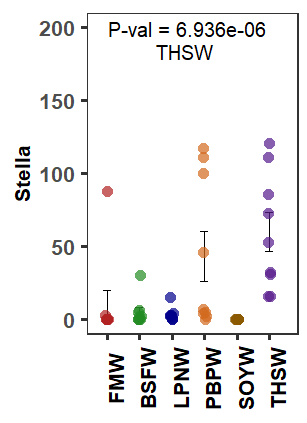

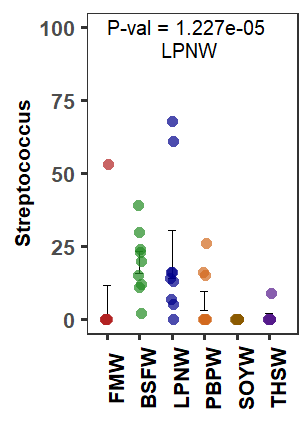

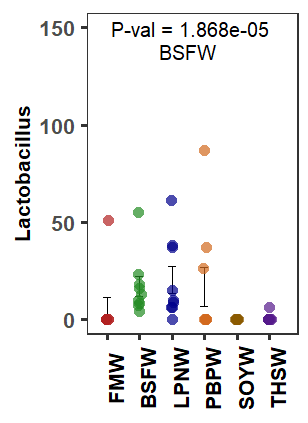


**Fig. 5**. Differential abundance of bacteria at genus level in the water under marron aquaculture fed different protein diets. Genera with more than 1% of read abundance in any of the group were used for statistical analysis.


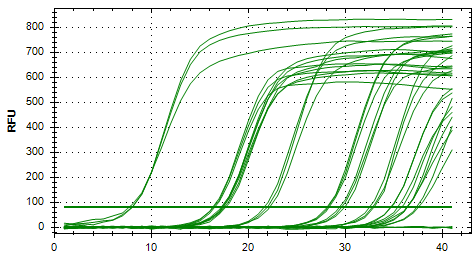


Soybean

Black soldier fly

Lupin

Feeding trial with six protein diets

Feeding trial with six protein diets


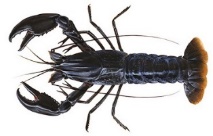

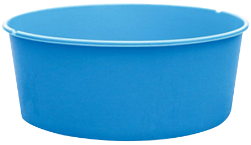


Fishmeal

Poultry-by-product

Tuna hydrolysate


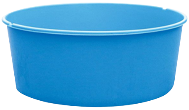

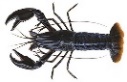

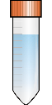


Sample collection after 60 days

Identification and quantification of gut and rearing water microbial communities


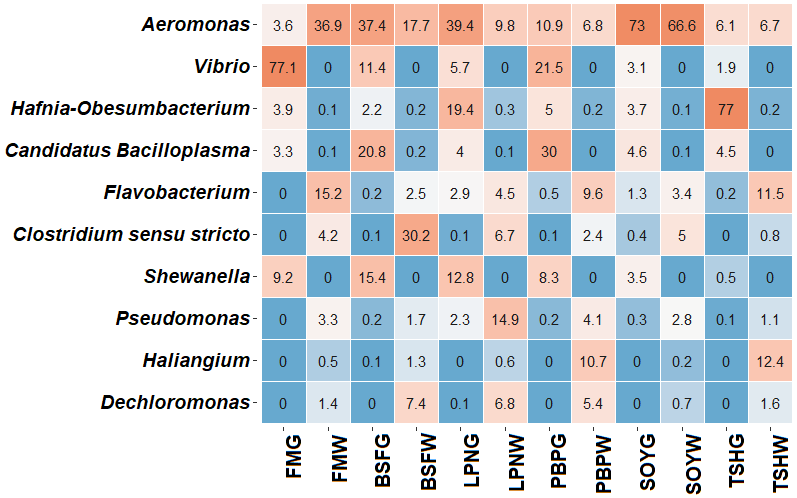

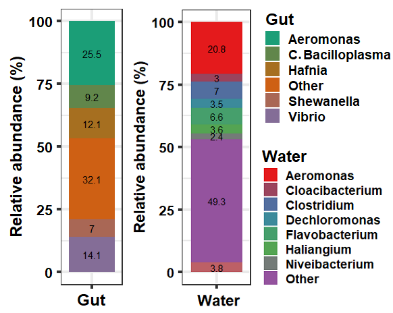


**Fig. S6**. An outline of experimental set-up and methodologies used in present study.

Table S1. Major diversity index for microbial communities in the gut and water

| Parameters | Water | Gut |
| --- | --- | --- |
| Reads | 3.0 million | 1.5 million |
| OTUs | 5731 | 745 |
| Unshared OTUs | 0 | 4986 |
| Phyla | 26 | 21 |
| Genera | 420 | 229 |

Table S2. Major diversity index for microbial communities in the gut with six different diets

| Parameters | FM | BSF | LPN | PBP | SOY | THS |
| --- | --- | --- | --- | --- | --- | --- |
| Reads | 27812.8±3052.1 | 22125±2626 | 25466±3056.3 | 20969.9±1990.7 | 23796.9±2675.6 | 25546.3±2046.9 |
| Observed | 112.2±29.8 | 117.0±33.8 | 128.8±9.7 | 144.3±33.8 | 121.6±18.1 | 85.1±16.8 |
| Shannon | 1.43±0.2 | 2.1±0.3 | 2.6±0.2 | 2.1±0.3 | 2.1±0.2 | 1.8±0.1 |
| Simpson | 0.6±0.1 | 0.7±0.1 | 0.8±0.1 | 0.7±0.1 | 0.7±0.1 | 0.7±0.1 |
| Chao1 | 228.8±74.6 | 141.0±35.9 | 178.9±54 | 189.6±34.6 | 160.8±22.5 | 132.6±23.7 |
| ACE | 225.5±75.2 | 141.3±34.6 | 178.0±15 | 191.7±32.7 | 163.8±22.5 | 141.8±26.9 |
